# Practices and values regarding milk consumption among pre-schoolers in Bangkok

**DOI:** 10.1080/17482631.2018.1461515

**Published:** 2018-04-18

**Authors:** Jomkwan Yothasamut, Laura Camfield, Michael Pfeil

**Affiliations:** a Health Intervention and Technology Assessment Program, Ministry of Public Health, Nonthaburi, Thailand; b School of International Development, Faculty of Social Sciences, University of East Anglia, Norwich, UK; c School of Health Sciences, Faculty of Medicine and Health Sciences, University of East Anglia, Norwich, UK

**Keywords:** Milk-consumption practices, pre-schoolers, sociocultural factors, values, Thailand, Bangkok Metropolitan Area, Ecological System Theory

## Abstract

**Purpose**: Thai government agencies and the business sector have been promoting milk consumption. Considering the robust and continual movements by those actors to promote milk consumption among children in Thailand at the national level, this study aims to investigate milk-consumption practices and values towards milk consumption at pre-school, family and individual levels. **Methods**: This cross-sectional qualitative study employs observation and interview methods, along with the Ecological System Theory as a framework. Data were collected from three kindergartens used by families of varying socio-economic status, and the homes of 18 pre-schoolers, aged 3-5 years old, attending these kindergartens, from October 2013-September 2014. **Results:** Findings reveal kindergartens implemented daily routines to make children drink milk. Practices at home include (i) overfeeding of milk, (ii) preference for fortified milk and (iii) using sweetness to make children drink milk. These practices were underpinned by values that milk is good for children and good parents feed their children milk. These values, in combination with other macro-level measures such as the government’s milk-promotion campaigns and the milk industry’s marketing, influence the milk-drinking practices of pre-schoolers. **Conclusion**: The promotion of the benefits of milk prompted children to exceed the recommended milk consumption of 400ml per day. Balanced information on moderation in milk drinking was absent.

## 1. Introduction

Studies of sociological factors that influence the food-consumption behaviours of young children have been carried out in the global North. For example, Kaufman and Karpati (2007) employed an ethnographic approach in order to understand the sociocultural roots of childhood obesity among Latino families in Brooklyn, U.S.A. A recent study by Elliott (2014) explored the perceptions Canadian teenagers (aged 12–14 years old) had of certain types of food, such as milk, broccoli and fast food and urged health promotion practitioners to pay careful attention to promotional messages of these food. There are few studies of such sociological factors in the global South. Chan, Deave, and Greenhalgh (2010) undertook an ethnographic study with 10 pre-schoolers, both obese and non-obese, in Hong Kong. They suggested that the main carers showed confusion as a result of mixed messages about proper health behaviour. In addition, external social structures including social values drive individual increases in consumption. There are no studies about the sociocultural dimensions of milk consumption among pre-school-aged children.

**Figure 1.**
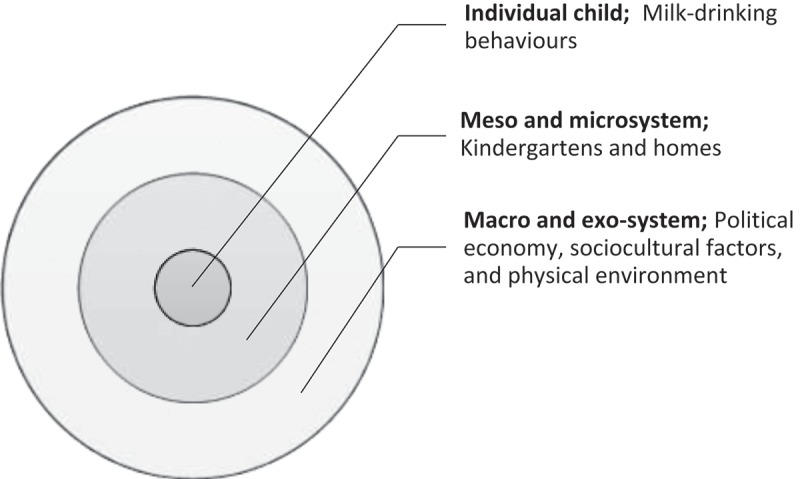
The application of EST in the study of the milk-drinking behaviours of pre-schoolers.

**Figure 2.**
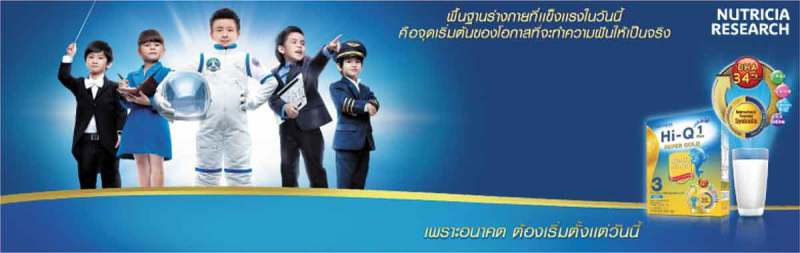
The advertising banner for a fortified milk brand

**Figure 3.**
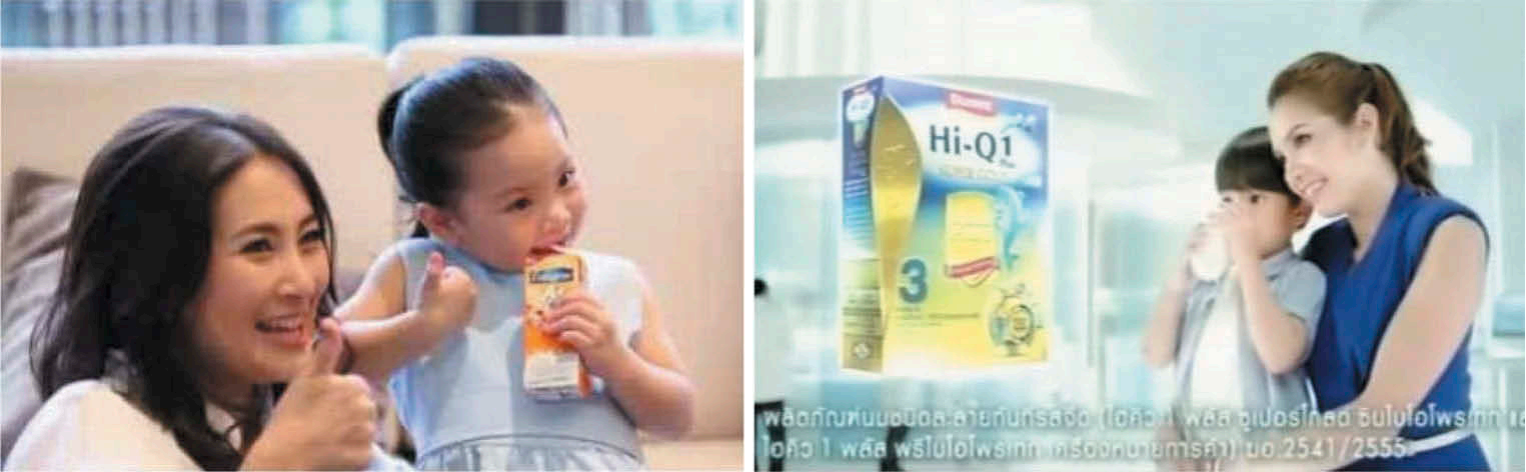
Screenshots from TV milk advertisements.

**Figure 4.**
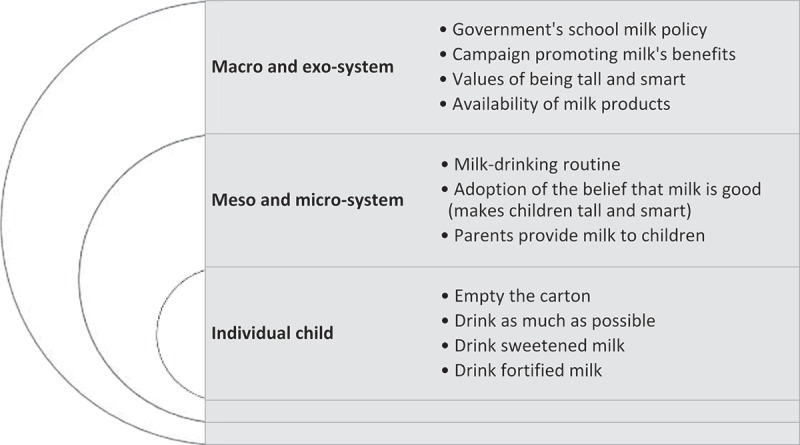
Factors from different ecological system levels that are linked to individual’s practices.

This study of values and practices regarding milk consumption among pre-schoolers in Bangkok employs the Ecological System Theory (EST) to guide an exploration of potential factors, from macro to micro levels, and behaviours that are nested. We begin this paper by introducing the context, that is, the history, policy and economic environment of milk promotion in Thailand, which are macro-level factors. This includes messages and interventions from actors in this system who communicate with individuals. The behaviour that we studied is milk drinking among pre-schoolers at kindergartens and in homes, which is discussed in detail in the methodology and findings sections. Our investigation includes milk-consumption practices at meso and micro levels, the practices of parents and teachers concerning giving milk to pre-school-aged children in Bangkok and the values that underpin these practices. Practices at meso level were those occur beyond household or micro level.

## 2. Context

In Thailand, dairy products, including milk, have not historically been produced and are not part of a traditional Thai diet; milk consumption used to be limited to upper class and South Asian communities in Thailand. In 1962 milk was introduced in Thailand as an opportunity for Thai farmers to invest in the then novel livestock industry and it provided an opportunity to replace some imported products with those that were produced locally (Knips, 2006). After the introduction of milk to the Thai market, the dairy industry faced a number of difficulties in stimulating demand, largely as a result of the cultural barriers to consumption, which caused over-supply of milk in the domestic market. As a result, the then government launched a number of campaigns to encourage milk consumption among Thai people, including a well-known campaign whose slogan was “Have you drunk milk today?”, a message that positioned milk as a daily essential foodstuff for Thais (Smitasiri & Chotiboriboon, 2003). Other measures included the provision of dairy cattle breeds to farmers and the use of import tariffs to protect domestic industry (Sukharomana & Chiarathivat, n.d.). However, the situation did not improve much and in 1984 farmers requested further support from the government (Suwanabol, 2005). In 1992, the most successful strategy to solve the problem of unsold milk was initiated; the “school milk policy” was created by The National Milk Drinking Campaign Board (NMDCB) as a way of “killing two birds with one stone” by saving the milk industry and providing nutrition for schoolchildren (Kammungkhun, 2009). The policy has been run by the Dairy Farming Promotion Organization of Thailand (DPO), a state-run dairy commerce chaired by the Ministry of Agriculture and Cooperatives (MoAC), that has been distributing free plain milk to Thai children aged 3–12 years old in all state-owned and some privately owned schools since 1992.

Thai government agencies have been promoting the consumption of plain milk for two reasons. The first is to boost the milk market, as stated in an FAO article written by the NMDCB’s president:

the principle [sic] objective of the National School Milk Programme is to support the Thai dairy industry, by providing an outlet for locally produced milk. By providing milk to the young at an early stage, will also through time developed [sic] a taste for milk and hence a market for the future (Suwanabol, 2005).

The second reason was to promote the health of the population. The Ministry of Public Health (MoPH) has expressed its ongoing concern about the problem of under-nutrition and children’s physical and cognitive development. Despite strong links between milk consumption by children and health outcomes for them, well-known milk-promotion campaigns were managed by the NMDCB (Chungsiriwat & Panapol, 2009), not the MoPH. On the other hand the MoPH’s credibility in the eyes of the public, as a professional health organization, has been used to support the milk-promotion movement. The Ministry of Education has played a passive role in the national campaign but took over the policy implementation (Kammungkhun, 2009). National milestone campaigns include a recent promotion of milk drinking among adults and young people—to increase the average height of the Thai population by the Department of Health (Hodal, 2013). The campaign “Give milk to the one you love” (*Rak Krai Hai Duem Nom*), and the establishment of World Milk Day on 1stJune, 2001, by the FAO have also played a role on popularizing the product in Thailand through the MoAC.

In addition to state actors, the dairy industry also participates in milk-promotion campaigns, with the aim of selling their products in the domestic market. The domestic dairy industry consists of 20 businesses (in 2007) including the DPO, the biggest producer with 20% of dairy milk market share. The main market for dairy products is for domestic consumption; Thailand’s dairy product imports are greater than its exports (Food intelligence center, 2012). Apart from joining the government’s national campaign to boost milk consumption, the domestic dairy industry also promotes and brings to the market sweetened and other specialized dairy products. It should be noted that the DPO also produces flavoured milk to sell in the market, as well as producing milk to supply the government’s school milk scheme. Other non-state actors include relevant businesses, such as the milk carton industry and hypermarkets in Thailand, who also participate in the milk-promotion campaign (Brandage-Thaicoon, 2014).

Milk consumption is promoted by two groups of actors (state and business) and two different core messages are used. There are two main promotional schemes: (i) promotion of milk by government agencies using a message that milk consumption is good and (ii) promotion by industry of flavoured milk and other milk products, e.g., chocolate milk, drinking yogurt or fortified milk, which is cows’ milk to which the producers add vitamins and other supplements such as DHA (omega-3 fatty acid), which doubles its price. Milk consumption has been presented to consumers as an important practice to make children healthy, tall, intelligent and, implicitly, economically successful (examples of the marketing slogans are presented in the following paragraph). Promotional measures have encouraged milk consumption among Thai people, especially among young children (Food Intelligence Center, 2012). The amount of milk consumption per child under the government’s school milk programme increased from 2 litres per child per year in 1988 to 23 litres in 2002 (Suwanabol, 2005). The recent Health Examination Survey 2008–9 reported that 56% of children aged 2–5 years old drink approximately one portion (200 ml) of milk every day, while 26% drink sweetened milk (National Health Examination Survey Network, 2011). The school milk and lunch programmes are ongoing interventions among other community-integrated programmes under the country’s Poverty Alleviation Plan. The plan contributed to a successful reduction in under-nutrition among children under 5 years old from 51% in 1980 to 20% in 1990 and below 10% in 2006 (Chavasit, Kasemsup, & Tontisirin, 2013).

Within the school milk programme, plain milk is the only product provided. However, in the domestic market, a more diverse range of milk products is available, including sweetened milk, fortified milk and drinking yogurt. These products contain substantial amounts of sugar, averaging 8 grams of sugar per carton (data taken from the nutrition label of a random sample of products). In 2011, the dairy product with the highest market value was drinking yogurt (Food Intelligence Center, 2012). Milk products are positioned as food that is specially designed for children, which is reflected through their packaging, e.g., smaller cartons with colourful patterns and children’s favourite cartoon characters on the packaging. They are promoted through TV advertisements, billboards, printed and electronic media, and promotional booths in places like shopping malls (where families spend time together) and kindergartens. Apart from promotional messages from the government’s campaigns, the industry emphasizes the message that milk makes children tall, and has introduced a radical message to society:—“Milk makes your kids smart and enables them to become successful as they grow older”. The leading milk companies that launched such advertisements on television include Dumex (Dumex Hi-Q), Enfagrow (Enfagrow A plus), and Dutch Mill (DMalt Triple 3+). The latest examples of the advertisements have been available on television and YouTube since 2012.

Although the advertising of infant formula is controlled by law, this does not apply to products aimed at pre-school-aged and older children. The lack of legal controls has left room for controversial actions such as the use of cartoon characters on milk cartons to attract children, or the organization of promotional events for milk products in kindergartens (Food and Drug Administration Thailand, 1979) and has seen the industry make implicit claims that milk products can make a child smarter and able to perform better academically. However the Food and Drug Administration Thailand (FDA), with support from non-profit and social organizations, announced in 2007 that they were considering improving the law to control advertising for snack and milk products for young children better (Academic Resource Center: Thai FDA, 2007). This proposal from the Thai FDA elicited responses from the milk and snack industries that such action would create huge losses to the Thai economy. However, up until now regulation has not been amended although the issue is still under review (being discussed and announced as a resolution in the National Health Assembly).

The increasing visibility of milk promotion, with confusing messages sent to the population, could result in adverse milk-consumption practices by individuals, e.g., consuming sweetened milk or consuming too much milk, yet this has rarely been raised as an issue of public concern. For example, the consumption of sweetened milk can lead to dental caries and obesity in young children (Harris, Nicoll, Adair, & Pine, 2004; Malik, Schulze, & Hu, 2006; Tinanoff & Palmer, 2000). A 180ml carton of full-fat milk contains 120 calories, nearly 10% of the daily requirement of a child aged 3–5 years (1,300 calories per day). Given that a child also obtains energy from other sources, such as meals and snacks, children should not consume more than two cartons (400–500 mL) of milk per day (Smitasiri & Chotiboriboon, 2003; Working Group on Food Based Dietary Guideline For Thai, 1998), otherwise they can take in too much energy, which can lead to the development of obesity when they grow older. Given the strong movement of actors and their interventions at macro level, this study aims to reveal and analyse the individual behaviours concerning milk consumption and practices that are nested with the macro-level factors, namely political and economic context and social values that we have presented and to further explore other factors such as sociocultural factors that can influence people’s behaviours.

## 3. Methodology

### 3.1. Study design and conceptual framework

We employed qualitative methods of observation and semi-structured interview, to investigate practices concerning milk consumption at the individual level, as these approaches offer insights into how people think and act that can contribute to the body of knowledge about factors that influence the development of childhood obesity.

We consider milk consumption as a social practice and observe patterns of milk provision and promotion to explain current milk-consumption practices. We used an inductive approach to develop themes from coded qualitative data, which helped to reveal the values behind practices. We adopted the definition of “values” from the belief system theory proposed by Grube at al. Values

are single beliefs and transcend objects and situations…and are cognitive representations of individual needs and desires and of societal demands on the other. They are translations of individual needs into socially acceptable form that can be presented and defended publicly (Grube, Mayton, & Ball-Rokeach, 1994, P. 155).

A study from Rokeach (1973) explained family security value (taking care of loved ones) is a terminal value that one would use instrumental values to achieve. In this study, such values were found among parents who were the main carers of pre-school children. We also employed a term “belief” to report what parents think about milk feeding practices for their children, a mean to achieve such values. Beliefs found in this study happened in relatively shorter period of time, for example the belief in goodness of fortified milk consumption.

We used Ecological System Theory (EST), developed by Bronfenbrenner (1974), to explore (sub)factors in the society, including family, community, economy and policy, that influence practices (milk consumption among pre-schoolers), as well as the values underlying them. This theory was also used by scholars such as Davison and Birch (2001) and Harrison et al. (2011) to understand the determinants of childhood obesity. The EST offers a framework to understand influential agents and structures in the lives of pre-schoolers by dividing the factors into levels of system. In Figure 1, the macro- and exo-systems refer to the culture and values of wider society, e.g., political, legal and economic structures and mechanisms. In addition, the system involves those who influence the child and their caregivers, e.g., a parent’s work environment and neighbourhood environment. In the micro- and meso-systems, children directly interact with teachers, siblings and peers, in addition to their parents. These interactions occur at home and at kindergarten. The use of EST helps us to understand how the observed milk consumption of pre-schoolers can be explained by influences from wider social factors, e.g., the political economy, the physical environment and sociocultural factors. Below we describe how the data were generated.

### 3.2. Data collection

Primary data were collected through observation over the course of a year (October 2013–September 2014) at three public and private kindergartens in Bangkok. Three kindergartens that families of different socioeconomic status used were purposively selected. Temple Side Kindergarten is a state-run kindergarten and available free of charge. Families who use this kindergarten are those of lower economic status, e.g., the parents work as daily wage labourers. New Market Kindergarten is a private kindergarten that offers care for children at a cost of 1,500 Thai Baht (THB) (or 30 GBP) per month. This tuition fee is equal to 10% of the minimum monthly salary 15,000 THB (or 300 GBP) per month of workers with a Bachelor’s degree. This kindergarten was selected to represent a kindergarten that families of middle-economic status use. Private Land Kindergarten is a private kindergarten that offers its service for 15,000 THB (or 300 GBP) per month, and represents a kindergarten for families of high-economic status.

The choice to differentiate the kindergartens on the basis of socioeconomic status was made as some studies have suggested that nutrition provision as a national policy can provide some benefits to disadvantaged students (Kristjansson et al., 2007). Particularly in the urban (metropolitan) environment, socioeconomic status also links to other factors such as educational background, migration, food choices, occupation and working and living conditions, which can all affect the development of obesity. In addition, the distinct characteristics of the Bangkok Metropolitan Area—where all the media and communication are well covered (meaning that people living in this area are likely to expose to promotional messages or campaigns that have been implemented), basic facilities are available, and with high influences from economic drivers—provide a good context for a case study of how adults and children living in such an environment interact with each other and with their environment and negotiate for their preferred choice (with regard to milk consumption). Moreover, National Health Examination surveys indicate a significant increase in obesity prevalence, especially in children in urban areas (Aekplakorn & Mo-Suwan, 2009).

In addition to the general observation within the kindergartens, after one month six children aged three to five years old were recruited (from each kindergarten, which made 18 children in total). Open-ended interviews with the selected children and their teachers and parents were conducted. Key questions used to open the conversation included “Could you tell me about your child’s daily activity?” “Could you tell me about food that your child eats daily?” For household data collection, unstructured observation was used. The researcher spent time during the day with the families at the places they usually went to, such as their houses, offices, shopping places or village playground or public swimming pool at various times of the day, ranged from 8 am to 6 pm, depending on families’ schedules. Two to four visits, one to four hours per visit, per family were performed to observe the child’s food practices and interview the family about them.

### 3.3. Data analysis

The data concerning the frequency and amount of milk consumption were obtained from kindergartens and the households of the children; in kindergartens, the milk-drinking times were set as part of a daily routine. At home, the data used to estimate milk consumption were derived from parents’ verbal reports of their children’s milk consumption. The researcher used specific questions to estimate children’s milk consumption: ‘can you tell me how much milk your child drinks before and after school time (or when they are with you)? and “what type and size of milk carton do you usually buy for him/her?”.

Data from the field were chronologically documented through field notes and recorded interviews (subsequently transcribed). The transcribed data were subjected to line-by-line data coding, from which themes emerged (this process was undertaken in Nvivo). JY was were the coder and LC and MP acted as peer-debriefer, ensuring accuracy and confirmability of the analytical results. The data focused on children’s food consumption and physical activities and the relevant factors and actors. Forty sub-themes (nodes) emerged from the data-coding, of which eight were grouped into the milk-drinking practice theme. The rest of the themes were about main meals and snack consumptions.

Values and beliefs related to milk drinking practice were used to guide and expand document and literature search and perform the analysis. Additional documents retrieved for analyses included advertisements of milk drinking available on television and electronic sources from 2005 to 2015.

## 4. Findings

### 4.1. Milk drinking practices

Out of the 18 children selected for further observation and interview, eight children consumed 400 mL of milk products, while seven children consumed more than 500 mL and three children consumed fewer than 200 mL per day. Two children drank up to 1,200 mL of milk products per day, substantially in excess of the recommended intake, and which represents 840 calories daily (up to two-thirds of their recommended daily calorie allowance). Both the feeding practices of parents and children’s food preferences contributed to the consumption practices. Overconsumption of milk, i.e., more than the recommended amount of 200–500 mL (130–330 calories) per day for children (Working Group On Food Based Dietary Guideline For Thai, 1998), was found in households across different socioeconomic groups. Milk products that the children drank ranged from standard plain school milk to fortified milk, sweetened milk and sweetened drinking yogurt. All children were given some kind of milk either at school or home. In addition, the three groups of families participating in the study, of different socioeconomic statuses, showed equivalent core values concerning milk feeding to children and sincerely cared about providing milk for their children. There were also a few cases of children who could not drink cow’s milk because of health conditions; however, these children were given alternative milk drinks, such as soya milk or special formula milk drink (for those from wealthy families). Milk consumption was mentioned by parents of all socioeconomic statuses and by teachers as an integral part of child rearing. The values that influence their careful practices of milk provision to the children have been revealed throughout the course of the investigation.

Kindergartens’ nutrition policies are designed to respond to parents’ and the government’s concerns about milk drinking. All kindergartens make milk drinking a routine activity akin to school lunches or other regular classroom activities. The amount or types of milk that children drink depend on the kindergarten’s policies. Public kindergartens like Temple Side Kindergarten have one milk-drinking time each day using plain milk from the government’s school milk scheme; meanwhile the two private kindergartens, New Market and Private Land kindergartens, scheduled two and three milk times respectively (these consisted of a combination of the government’s free milk and milk that parents provided for their children, which was usually fortified and/or flavoured). The frequency of milk time also depends on the kindergartens’ operating times (the hours that children spend in kindergartens), ranging from 1 to 3 times per day. In kindergartens children would drink between one and three portions of milk (180–250 mL and 540–750 mL, respectively). However, children might or might not have drunk more milk when they were at home.

The milk feeding practices of teachers may not guarantee that they (as individual adults in the society) embrace the value of providing the best (milk, in this case) to children. Overfeeding of milk in kindergartens can be because the teachers want to obey the kindergarten’s director/owner. Likewise, the policy of the kindergartens’ owners might aim to please parents, the customers of the kindergarten. Nonetheless these feeding practices are associated with the fact that people in the society know that parents want their children to drink milk (to provide the best to their children).

Bearing in mind the continual movements through national policy, promotional messages and marketing activities regarding milk consumption at macro level, the next sub-section presents findings from a cross-sectional study investigating values and beliefs concerning pre-schoolers’ milk consumption.

### 4.2. Values and beliefs underlying the practices

The values and beliefs concerning the milk consumption practices, including the selection of milk products, purchasing milk, and feeding it to children, of pre-schoolers that have been revealed through the analysis and include: “milk is good for you: it makes children grow smart and tall”, “fortified (expensive) milk product makes children smart”, and “give milk to the one you love”. In this section, we use these values and beliefs to elaborate our findings. It is noteworthy that these findings are based on investigation within three kindergartens in the Bangkok Metropolitan Area. This area is more economically developed than other parts of the country.

Adults’ positive perceptions about milk consumption were reflected in the practices and expressions of the parents in our sample. These included their emphasis on managing their children’s milk provision and consumption during an interview with open-ended questions about their children’s daily activities and food. Parents of the 10 children who had no problem drinking plain milk told me proudly how much their children liked to drink milk, opening the carton themselves and drinking it without being forced; meanwhile parents of children who did not like milk explained how hard they worked to find milk that they would drink. The teachers at the three kindergartens reported on the food and milk consumption of the children to their parents verbally or in the kindergarten logbook on a daily basis.

In addition to individual expressions and practices, the mass media carries messages developed by government agencies and the milk industry promoting the consumption of milk by Thai people of all ages (Food Intelligence Center, 2012).

#### 4.2.1. Milk is good for you: it makes children grow smart and tall

Believing that milk is best for their children, many parents encouraged them to drink as much as they could and even replaced water with milk drinks, leading to overfeeding. Minnie, aged three and overweight, attends Private Land Kindergarten. Her mother who has a food science background said:

Minnie drinks a lot of milk. We let her drink milk instead of water … so in total … she drinks a litre at home, plus two bottles (120 ml each) at kindergarten. You know, we only choose pasteurised [full fat] cows’ milk for her as it is the best and most nutritious option compared to UHT milk. (Interview of Minnie’s mother, August 2014)

Minnie’s case illustrates the care that parents take over both the quality—selecting fresh rather than UHT milk—and the quantity of milk drunk, e.g., replacing water with milk. This case proves the success of advertising and campaigns in the Thai population. The parents talked proudly of their careful selection of the best milk and the big amount of milk they could encourage their children to drink. Minnie was one of the children in our study whose parents supported and encouraged them to consume products that they believed to be beneficial, such as full fat fresh milk. They were unaware of the guidelines on limiting young children’s milk consumption. For example, when parents were asked whether they knew how much milk they should give their children only one mother reported that she had learned from an article somewhere that she should not give her son more than two cartons (equal to 400 mL), according to the guidelines.

This perception is supported by a strong promotional government message with support from the milk industry through advertising. Since the introduction of milk to the Thai population in 1962 with the establishment of a domestic milk industry, the Thai government has launched a number of campaigns to encourage drinking milk including a well-known one with the slogan “Have you drunk milk today?,” a message positioning milk as an essential daily food (Smitasiri & Chotiboriboon, 2003). This was followed by a number of campaigns and the recent Department of Health’s promotion of milk for adults and young people to increase the average height of the Thai population (Hodal, 2013). Through the establishment of World Milk Day on 1st June 2001, the Food and Agriculture Organization of the United Nations (FAO) has also played a role in popularizing the product in Thailand in conjunction with MoAC. Apart from the government’s promotional messages and the international support, the industry emphasizes the message that milk makes children grow tall, and has introduced a radical message to society together with new milk products on the market.

Good cognitive (i.e., being smart)and physical development (i.e., being tall) are the main concepts that Thai people value as desirable in their children, as shown in studies by Hesse-Swain (2006). These values were also found in the parents in this study. The way they most often encouraged their children to drink milk was by telling them that it would make them grow tall. “For milk, I don’t have to order him to drink, he grabs the carton and drink by himself. He wants to be tall,” Aim’s mother told me during an interview. The children who liked to drink plain milk had absorbed this concept and explained it to me during my observation and interview with them. It is certain that young children can easily understand the concept of being tall, and it also fits with what many children want. Parents in this study believed that milk would give their children health benefits, including good nutrition to make them healthy and support their growth and development. These beliefs were expressed at the interviews and in their practice of feeding their children milk. Being smart is related to skills development, which in later life will lead to economic success for each individual; meanwhile the notion of being tall is equated with being good-looking. Being tall is one of the norms in the conception of beauty in Thailand (along with fair skin, an oval face, an angular and narrow nose and wide eyes) defining the “international look” (Hesse-Swain, 2006). With respect to these values, appealing promotional messages have been communicated via the government’s milk-promotion campaign and the business sector’s advertising. The government’s campaign and announcement from the MoPH emphasize children’s physical and cognitive development and encourage people to drink milk, but do not imply that this will lead to success as an adult, as industry advertisements do.

#### 4.2.2. Fortified (expensive) milk product makes children smart

Fortified milk is promoted as the best milk drink, with the industry claiming that it fortifies its milk with a number of vitamins and nutrients. The formula and fortified milk market has been growing continuously since 2010, partly because of stronger controls on the marketing of formula milk, extensive promotion of breastfeeding and the control of sugar in formula milk. Advertisements from milk companies in the mass media find ways of sending the strong message that their product can make children smart—one advertisement uses as a pre-school child as their product presenter who drinks this milk and is the best academic performer in the class. Such advertisements do not claim direct benefits, but their design could lead consumers to form such an impression. For instance, an advertisement starts by describing the scientifically proven benefits of DHA and vitamins and then continues to describe how a company’s milk product also includes these nutrients, followed by a scene with the child who drank the product in a previous scene answering all the questions the teacher asks in the classroom. Without making any direct claims, all the presentations and sequences of the advertisement are designed to lead audiences to the conclusion that the product makes children smarter. As parents want to provide the best for their children, this includes providing what they perceive to be the best milk, and the milk producers’ marketing messages resonate well with this.

Another example of an advertisement for a brand of milk states: *“Today’s healthy body creates opportunities to make dreams come true…because the future starts today”*, hinting that milk makes your children smart and enables them to become successful as they grow older. Figure 2 shows children in costumes related to prestigious careers to imply cognitive development that will create future success, mostly represented by scientific careers such as astronaut and scientist. These macro-level interventions (including campaigns and policies such as the school milk programme) aim to create positive perceptions of drinking milk in adults. The leading milk companies that run such advertisements on television include Dumex (Dumex Hi-Q), Enfagrow (Enfagrow A plus), and Dutch Mill (DMalt Triple 3+). The latest examples of the advertisements have been available on television and YouTube since 2012.

The use of symbols to link milk products and brands with knowledge and academic performance is popular, especially in the case of fortified milk products. The two leading brands use the terms A+ and Hi-Q to brand their products. A+ implies the highest academic achievement, while Hi-Q refers to a high IQ or cognitive development. The nutrients that these products usually add to the milk include iodine and omega 3 (DHA), which, it is claimed, help in brain development. Other nutritional supplements that the companies add to their products include vitamins A, B12 and C, calcium, phosphorus and zinc.

These products were popular not only among families with high socio-economic status but also among the low socio-economic group. Parents and other caregivers tried their best to seek out such products for their children even if their resources were limited, because they believed that they provide better benefits. The grandmother of Bright, an overweight four-year-old boy attending Temple Side Kindergarten, said: *“I can feel he’s smarter when drinking those expensive milk products, as they put a lot of vitamins and nutrients into them.”* Then she turned to her grandson and said, *“Now you need to drink cheap [free school] milk for a while, my boy. We’ve run out of the expensive ones.”*

The quote from Bright’s grandmother reflects a strong combination of values and influences from milk industry advertisements. Furthermore, it shows that even though the family had limited resources it still provided these expensive milk products. The provision of expensive fortified milk to pre-schoolers is becoming very popular, with the highest market value of 2.5 billion baht in 2014, while sales of plain cows’ milk, soya milk and drinking yogurt totalled 1.4 billion, 900 million and 1 billion baht respectively (“Expand UHT”, 2015). At Private Land Kindergarten most of the shelves where the children’s milk is kept are filled with this type of fortified milk. Meanwhile parents of lower economic status made efforts to purchase these products for their children when they had the resources to do so.

The Thai goal of bringing up a child to be smart and successful with good academic performance has become popular among parents, especially in urban areas (Tapanya, 2011). All the teachers and parents in this sample observed that the requirements of young children’s academic performance has increased considerably compared to their own generation. Sending children to tutoring schools has become a popular practice. From our observation at Private Land Kindergarten, children attended six lessons a day; these included English and Mandarin for children aged 2–5, mathematics for 4–5-year-olds, and cognitive skills development. This mostly happens at private kindergartens, where the parents are on a higher socio-economic level, while buying expensive milk is an accessible choice for those cannot afford to send their children to tutoring schools. The teachers said that they had to teach more difficult lessons to the children to enable them to compete with others in the entrance examinations for the famous primary schools organized by leading government universities. This also enhances the reputation and guarantees the academic quality of the kindergarten concerned.

We observed little differences in milk consumption between children from families with high, middle and low-economic status. This is also because the children received free milk at kindergarten under the Thai government’s School Milk Policy. Families on a limited budget may stop buying “expensive/fortified milk” products when they run out of money and rely on the free school milk. Meanwhile those with higher-economic status can purchase fortified milk without constraint. Families on a limited budget showed us how they flavoured plain school milk by adding sugar to make it more palatable to the child. This suggests a need for better public education about milk and sugar consumption among pre-schoolers, targeting parents and other adult carers.

#### 4.2.3. “Give milk to the one you love”

Considering all the perceived benefits of milk, parents tried to provide this nutritious drink for their children. This was clear on our visits to the children’s families. The parents tried to get both large amounts and the best quality of milk for their children. If they did not like milk, the parents focused on giving them whatever milk they would drink, including sweetened products.

The parents’ perception that they should feed their children milk led them to employ a number of strategies to persuade the children to drink it. The commonest example was parents negotiating with their children and ending up offering them a sweetened milk drink. None of the parents or teachers expressed concern or knowledge about the possible negative effects of overconsumption of full-fat milk, or doubts about the benefits claimed by fortified milk producers. Only a few parents knew about the drawbacks of drinking sweetened milk, as illustrated by the case of Aim, who had not been breastfed as a baby. His mother said *“He was fed with powdered milk since he was born. When he was [2–4 years old] and was still on a bottle, I would prepare four bottles and leave them in his bed, and he would grab the bottles and finish them during the night. This is why he got caries”*. However, parents’ beliefs about the benefits of milk and their desire to provide good things such as milk for their children led them to ignore such potential harm, e.g., Aim’scaries; and Neutron, whose mother was advised by her son’s paediatrician that she should only give plain milk, to her son, who refused to drink it but was happy with sweetened milk. Thus the parents decided to let their children continue drinking sweetened milk, as they believed that at least they were drinking some form of milk.

This perception can be linked back to the “Give milk to the one you love” (*Rak krai hai duem nom*) campaign, one of the most popular since early milk promotion in the country. The promotional message was followed by a series of TV advertisements produced by the milk industry presenting scenes with parents taking care of their children. Advertisements for children’s milk commonly use pictures and scenes of mothers offering milk to their children accompanied by messages emphasizing the love that the mothers are showing for their children by giving them milk, as illustrated in Figure 3.

## 5. Discussion

Multiple factors from different levels of the ecological system contributed to the milk-drinking practices of individuals (see Figure 4). Certain drinking practices found in the study prompted the need to be aware of influences from factors at the macro-system level, e.g., government campaigns and advertisements, on individuals’ practices. Over-feeding of plain (full-fat) milk, the use of sweetness as a way to make children drink milk, and the preference for fortified milk products that claim to offer additional nutrients are practices of concern. The data from the observation suggested that drinking fortified milk had become a norm in certain sections of society; the shelves at the two private kindergartens were full of fortified milk, half of the parents indicated they provided fortified milk for their children, and such products are widely available in all supermarkets including 7-eleven convenience stores. This is a concern because such practices are likely to lead to the development of health problems such as obesity and dental caries in young children (Harris et al., 2004; Malik et al., 2006; Tinanoff & Palmer, 2000). These practices occur both at kindergartens, where milk drinking is part of the daily routine (with teachers ensuring children drink up the provided milk), and in homes.

Parents and teachers reflected that it was their role to encourage children to drink milk according to the presented values underlying milk feeding practices found in this study. These values influence the development of milk feeding and consumption practices. The first two values influenced parents’ feeding practices, which resulted in the overfeeding of milk and the provision of specialized milk products, such as sweetened and fortified milk products, to pre-schoolers.

The cognitive development (to be smart) and the physical development (to be tall) of children are the main concepts that Thai people valued as desirable. These values were also found from parents in this study. The concept used most often by adults in this study to encourage their children to drink milk was greater height. Being smart is related to skill development which in later life will lead to economic success for an individual person; meanwhile the notion of being tall hinted that the person was good-looking. Being tall is one of the norms in the conception of beauty in Thailand (along with fairer skin, an oval face, a more angular and narrow nose and wide eyes) to define the “international look” (Hesse-Swain, 2006). With respect to these values, promotional messages have been communicated via the government’s milk-promotion campaign and the business sector’s advertising that appeal to them.

Meanwhile messages about the negative consequences of milk drinking, e.g., that milk will make you fat if you drink too much or sweetened milk will create dental caries, were poorly communicated. It is a critical time to reflect on the implications of the development in milk-consumption patterns in the country, bearing in mind that government agencies continually promote the consumption of milk with the aim of increasing domestic consumption in the near future by using figures for milk consumption by populations of other countries, including European and other Asian countries, as a comparative standard for milk consumption among Thai people (The Information and Public Relations Office, 2014). The evidence from this and other studies (Aekplakorn & Mo-Suwan, 2009; Elliott, 2014; Food Intelligence Center, 2012; Harris et al., 2004; Malik et al., 2006) suggests that the MoPH should reduce its promotion of the benefits of milk consumption and shift to an emphasis on the types of milk (i.e., plain milk) and recommended quantities of it. The public are susceptible to misinformation, especially around fortified milk, promoted by the dairy industry, and the Thai FDA has a responsibility to regulate and monitor the kinds of health information provided to the consumers at whom the promotions are targeted. In addition, other interventions, such as regulation or taxation, might be considered as a way of controlling sweetened and fortified milk products. The control of sugar adding in milk products could be incorporated with the current movement in 2014 from health policy researchers, networks of health professionals and NGOs to work with the Excise Department to implement taxation based on the amount of sugar in sweetened drinks. Currently certain sweetened drinks, e.g., sweetened milk and green tea, are under the tax exemption scheme.

In this study, the practices that we are interested in are “milk consumption among pre-schoolers” which is linked with adults’ practices. This is because pre-schoolers rely on adult carers. Although children’s decisions and actions can be influenced by many factors, the very powerful factor that shapes the milk-drinking practices of children is the daily routine that is set at kindergarten and the organization of the home environment according to the values and beliefs that parents (adult carers) hold. These influence the choices and actions of the children.

The insignificant differences in milk consumption between children from families of high, middle and low economic status is partly because of the free milk support that children received from kindergartens. Families with limited budgets may stop buying “expensive/fortified milk” products when they run out of budget and rely on the free school milk. Meanwhile families of higher economic status can purchase fortified milk without any constraints. In addition, families with limited budgets showed how they flavoured the plain school milk by adding sugar to make the milk more favourable for the child. This suggested a need for better public education about milk consumption among pre-schoolers (targeted at parents and other adult carers).

Further research should be performed to provide evidence on the benefits of the dairy milk promotion scheme, i.e., the economic benefits for farmers and health benefits for the population. Any recommendations should be evidence-based by using the existing national health profile data to locate current health problems, including under-nutrition, obesity, and child growth and development and deliberating how milk consumption as an intervention should be effectively designed and used to tackle those problems and in what settings.

The use of EST to investigate and understand factors that influence milk consumption practice, as in this study, can be applied to similar issues, such as the investigation of factors that influence other food practices. For instance, researchers could study meal and snack provision for children, which can influence development of childhood obesity, a complicated problem and increasing in the global South.
